# AMPK activation by AICAR sensitizes prostate cancer cells to radiotherapy

**DOI:** 10.18632/oncotarget.26598

**Published:** 2019-01-22

**Authors:** Colin Rae, Robert J. Mairs

**Affiliations:** ^1^ Radiation Oncology, Institute of Cancer Sciences, University of Glasgow, Glasgow, UK

**Keywords:** prostate cancer, radiosensitizer, AMPK, AICAR

## Abstract

Although radiotherapy is often used to treat localized disease and for palliative care in prostate cancer patients, novel methods are required to improve the sensitivity of aggressive disease to ionizing radiation. AMP-activated protein kinase (AMPK) is an energy sensor which regulates proliferation, aggressiveness and survival of cancer cells. We assessed the ability of the AMPK activator 5-aminoimidazole-4-carboxamide-1-β-D-ribofuranoside (AICAR) to sensitize prostate cancer cells to radiation. Prostate cancer cell lines LNCaP and PC3 were treated with X-rays and AICAR then assessed for clonogenic survival, spheroid growth delay, cell cycle progression, and AMPK and p53 activity. AICAR synergistically enhanced the clonogenic killing capacity, spheroid growth inhibition and pro-apoptotic effect of X-rays. The mechanism of radiosensitization appeared to involve cell cycle regulation, but not oxidative stress. Moreover, it was not dependent on p53 status. Treatment of PC3 cells with a fatty acid synthase inhibitor further enhanced clonogenic killing of the combination of X-rays and AICAR, whereas mTOR inhibition caused no additional enhancement. These results indicate that interference with metabolic signalling pathways which protect cells against irradiation have the potential to enhance radiotherapy. Activation of AMPK in combination with radiotherapy has the potential to target metabolically active and aggressive tumors which are currently untreatable.

## INTRODUCTION

Prostate cancer is the most commonly diagnosed cancer amongst men and the second most common cause of cancer death in men [1]. It is a disease that predominantly affects men over the age of 50 and its incidence is expected to increase. Although radiotherapy is often used for control of localized disease and palliation, resistance is common and there is no effective treatment for disseminated disease [2]. Treatment options for patients with metastatic, relapsing and castration-resistant disease are currently limited to hormonal manipulation and radical prostatectomy. Therefore, new strategies which exploit the inherent differences between tumors and normal cells are required to sensitize tumors to radiation [3].

A hallmark of malignancy is abnormal metabolism which generates biomass and energy for proliferation, migration and cell signalling. This metabolic adaption by cancer cells also confers survival advantages to these cells and contributes to resistance to therapy [4]. Increased lipogenesis is observed in prostate cancer cells and is associated with their rapid growth and aggressiveness and inhibitors of fatty acid synthase (FASN) reduce cancer cell proliferation [5, 6]. Compared with their radiosensitive counterparts, radioresistant cancer cells also have altered components of energy metabolism, including elevated levels and activity of FASN [7, 8]. Furthermore, in response to exposure to ionizing radiation, metabolic regulators such as 5’ adenosine monophosphate-activated protein kinase (AMPK) are activated [9].

AMPK, a signalling protein activated by energetic stress, is a key energy-sensor whose activation by increased AMP:ATP ratios leads to restoration of energy homeostasis by switching on catabolic pathways thereby generating ATP, while switching off ATP-consuming processes [10]. As these anabolic pathways include cell growth and proliferation, there exists a possibility that activation of AMPK may be beneficial in cancer therapy. Indeed, it has been demonstrated that activation of AMPK by the AMP analog 5-aminoimidazole-4-carboxamide-1-β-D-ribofuranoside (AICAR) inhibited growth of prostate cancer cells [11]. Importantly, the increase in apoptosis and reduction in cell viability and proliferation observed in cancer cells treated with AICAR were not observed in non-cancer cells, indicating that this drug exhibits preferential toxicity for cancer cells [12–14]. AICAR is a cell-permeable nucleoside which is converted intracellularly by adenosine kinase to AICAR monophosphate which binds the regulatory γ subunit to activate AMPK. Novel drugs which directly activate AMPK also decreased proliferation, increased apoptosis of cancer cells and inhibited tumor growth in animal models [15]. Furthermore, drugs which act indirectly to activate AMPK, including metformin, aspirin and FASN inhibitors, have also been demonstrated to decrease proliferation of cancer cells [6, 16–18] and potentially sensitize cancer cells to radiation [8, 9, 19, 20]. The anti-tumor effect of activation of AMPK has been postulated to occur through several distinct mechanisms, including inhibition of two major drivers of prostate cancer carcinogenesis, lipogenesis and the mTOR signalling pathway [21, 22], indicating that AMPK activators may offer an advantage over drugs targeting only one anti-tumor pathway. Therefore, our aim was to evaluate the potential radiosensitizing effect of AMPK activation on prostate cancer cells and determine possible mechanisms of interaction.

## RESULTS

### AMPK activation is toxic to prostate cancer cells

**Figure 1:**
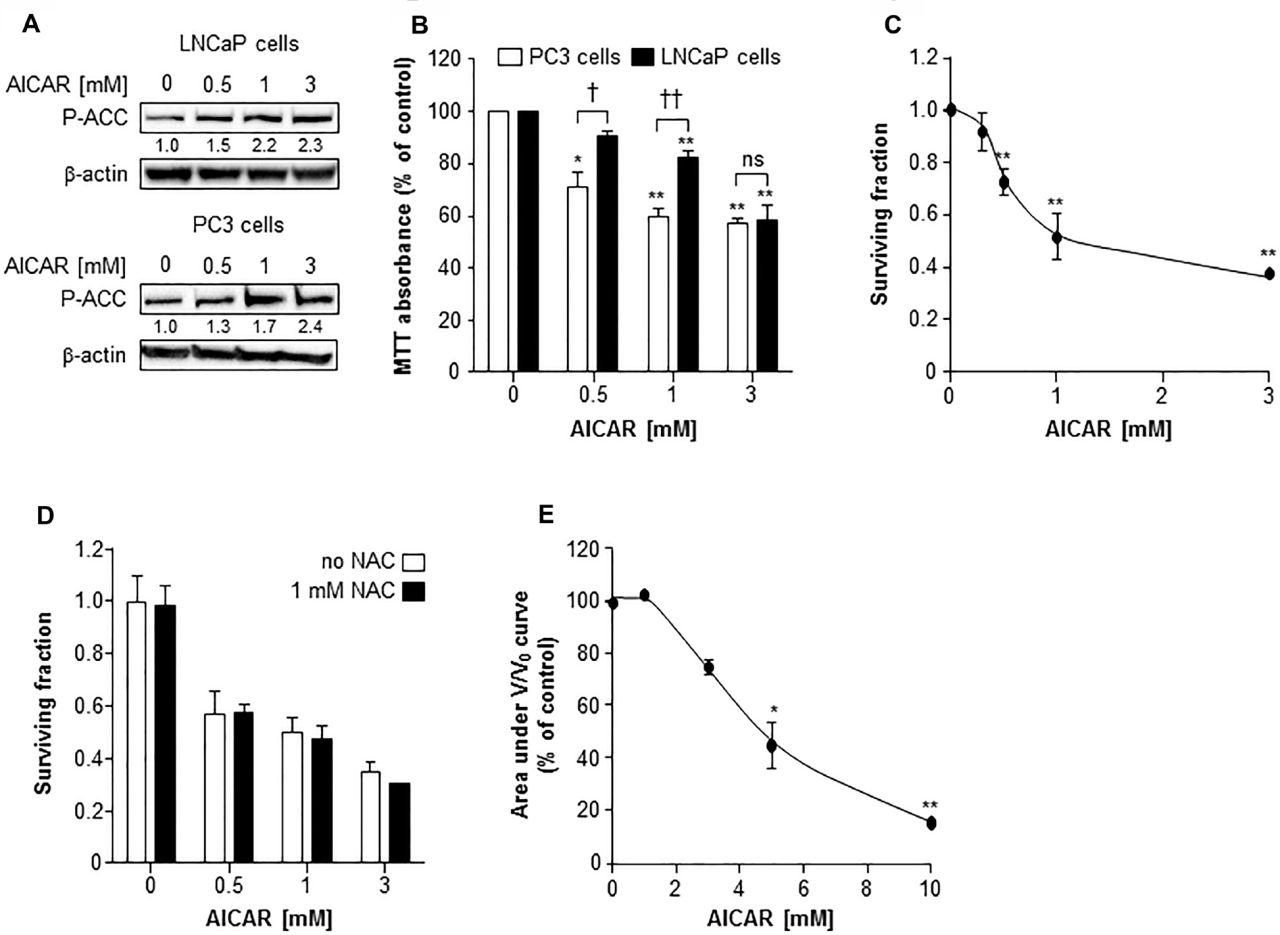
The cytotoxic effect of AICAR as a single agent on LNCaP and PC3 cells (**A**) Expression of phosphorylated acetyl Co-A carboxylase (P-ACC) in LNCaP cells 6 h and PC3 cells 24 h after administration. Average fold change in expression relative to control is shown under blot, mean of 3 separate experiments. β-actin is used as a loading control. (**B**) MTT assay of PC3 (white bars) and LNCaP (black bars) cells 24 h after administration of AICAR. (**C**) Clonogenic survival of PC3 cells treated with AICAR for 24 h. (**D**) Clonogenic assay was also carried out in the absence (white bars) and presence (black bars) of NAC (1 mM), which was co-incubated with AICAR for 24 h. (**E**) Growth of spheroids composed of LNCaP cells was measured for 21 days after administration of AICAR for 24 h. Graph shows the area under the V/V_0_ curve. Data are means ± SEM, *n* = 3. ^*^p < 0.05 and ^**^p < 0.01 compared to untreated controls, ^†^*p* < 0.05, ^††^*p* < 0.01 and ns = not significant compared to other cell line treated with same concentration of AICAR.

AMPK activation by AICAR (0.5 to 3 mM) was manifest in LNCaP cells and PC3 cells by phosphorylation of acetyl Co-A carboxylase (ACC), a downstream target of AMPK (Figure 1A). Using the MTT assay to determine toxicity of AICAR to PC3 and LNCaP cells in monolayer cultures, it was observed that AICAR caused a concentration-dependent decrease in survival after treatment for 24 h (Figure 1B). AICAR was significantly more toxic to PC3 cells than LNCaP cells at concentrations of 0.5 and 1 mM. No further increase in cell kill resulted from treatment of PC3 cells with 3 mM AICAR, whereas LNCaP cells succumbed in a manner similar to that observed for PC3 cells. AICAR also induced a concentration-dependent decrease in survival of PC3 clonogens 24 h after administration (Figure 1C). The IC_50_ value was 1 mM. Simultaneous administration of AICAR with the antioxidant N-acetyl cysteine (NAC) indicated that the clonogenic kill caused by AICAR in PC3 cells was not dependent on oxidative stress (Figure 1D). Unlike PC3, LNCaP cells did not form clonogens, and so the effects of AICAR on these cells was assessed in spheroid growth assays. Conversely, PC3 cells do not form spheroids. As a single agent, AICAR also reduced growth of spheroids composed of LNCaP cells in a concentration-dependent manner (Figure 1E). Although the shape of the concentration-effect graph is similar to that of PC3 clonogenic survival, a higher concentration of AICAR was required in spheroids.

### AICAR sensitized prostate cancer cells to X-radiation

**Figure 2:**
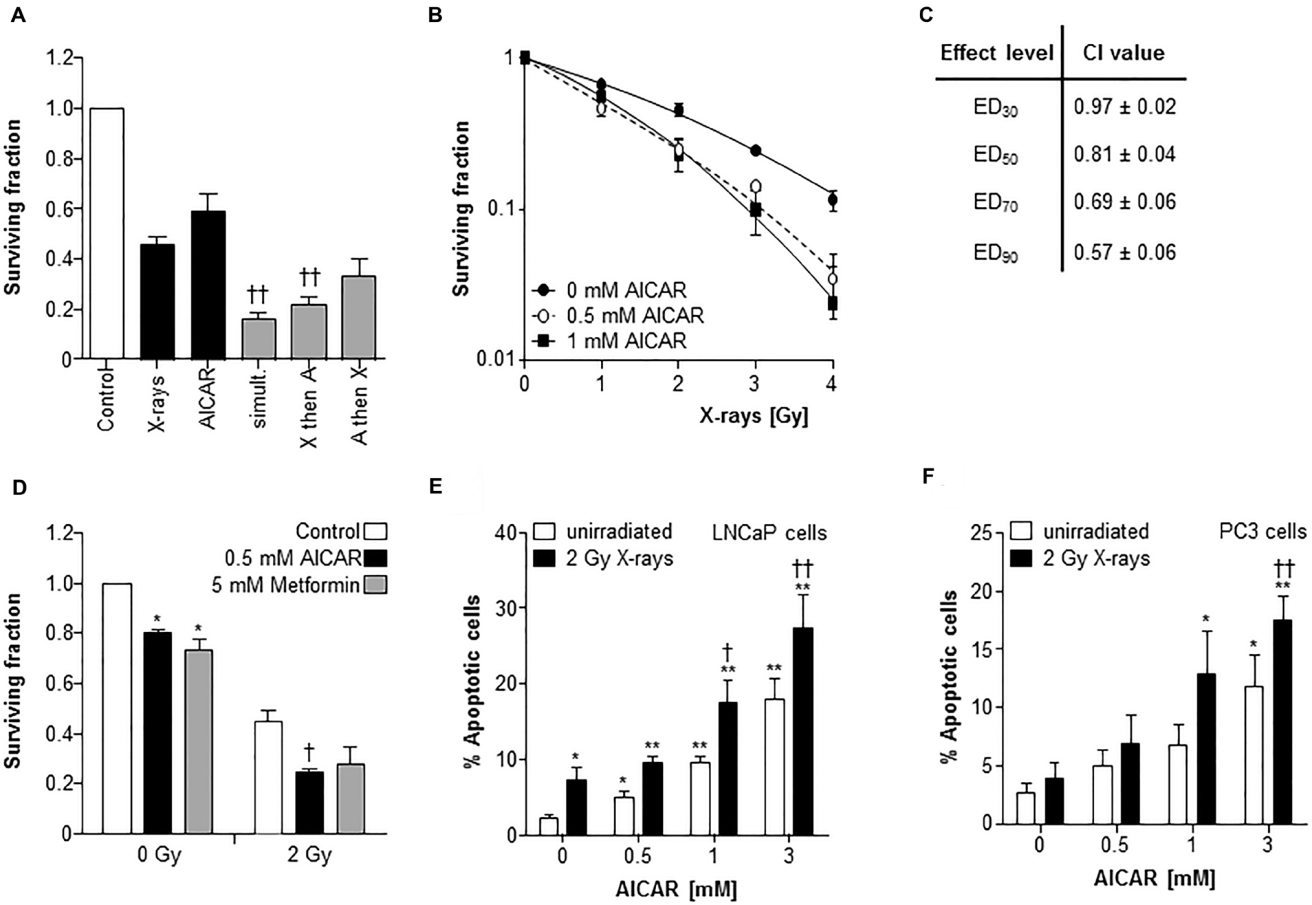
AICAR sensitizes PC3 cells to experimental radiotherapy (**A**) The effect of administration schedule of the combination of AICAR (1 mM) and x-radiation (2 Gy) on the kill of PC3 clonogens was tested using 3 administration schedules (i) radiation and drug administered simultaneously, (ii) radiation administered 24 h before drug, (iii) radiation administered 24 h after drug. (**B**) Radiation kill curves of PC3 cells exposed to AICAR (0.5 or 1 mM) and x-radiation at a range of doses, administered simultaneously. (**C**) The effect of treatment of PC3 cells with AICAR and x-radiation on combination indices. CI values are mean ± SEM of 3 experiments. EDx = dose required to kill x% of clonogens. (**D**) The effect of AICAR (0.5 mM) or metformin (5 mM) on clonogenic survival of PC3 cells exposed to 0 or 2 Gy x-irradiation. Effect of single agents and combination treatments on apoptosis (% of propidium iodide-stained cells in sub-G_1_ phase) 24 h after simultaneous administration of AICAR and radiation (2 Gy X-rays) on (**E**) LNCaP and (**F**) PC3 cells. Data are means ± SEM, *n* = 3. ^*^p < 0.05 and ^**^p < 0.01 compared to untreated controls, ^†^*p* < 0.05 and ^††^*p* < 0.01 compared to radiation treatment alone.

**Figure 3:**
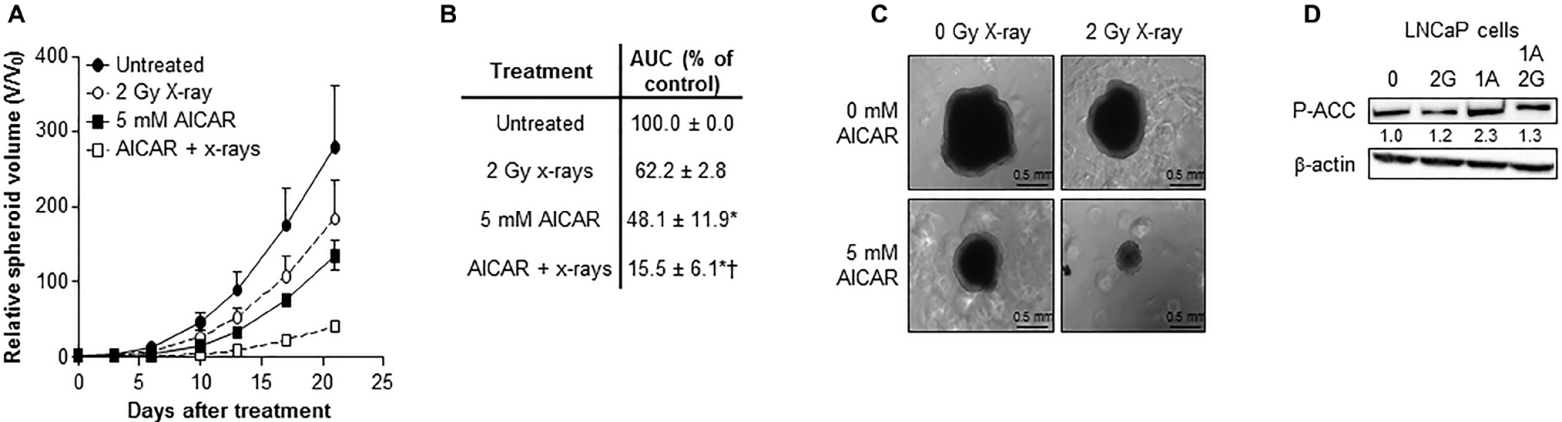
Combination of AICAR and ionizing radiation in LNCaP cells Growth of spheroids composed of LNCaP cells after simultaneous administration of AICAR (5 mM) and x-radiation (2 Gy). Data is presented as (**A**) relative spheroid volume over 21 days and (**B**) area under the V/V_0_ curve relative to untreated control spheroids. Data are means ± SEM, *n* = 3. ^*^p < 0.05 compared to untreated controls, ^†^*p* < 0.05 compared to radiation treatment alone. (**C**) Representative images of spheroids 21 days after treatment. (**D**) Representative blot of the effect of X-rays (2 Gy) and AICAR (1 mM) on phosphorylation of ACC. Average fold change in expression relative to control is shown under blot, mean of 3 separate experiments. β-actin is used as a loading control.

A comparison of the potency of alternative schedules of administration of the modalities AICAR and X-rays revealed that the most effective kill of PC3 clonogens was achieved when treatments were administered simultaneously (Figure 2A). Therefore, all further experiments utilized this administration schedule. After simultaneous administration, AICAR enhanced the clonogenic kill of PC3 cells induced by a range of doses (1 to 4 Gy) of radiation (Figure 2B). The surviving fractions following radiation treatment at a dose of 2 Gy (SF2) were 0.45 ± 0.03, 0.30 ± 0.02 and 0.25 ± 0.04 for 0, 0.5 and 1 mM AICAR, respectively, giving dose enhancement ratios (DER) of 1.86 ± 0.15 and 2.09 ± 0.31 for 0.5 and 1 mM AICAR, respectively. Moreover, combination index analysis (Figure 2C) indicated that at all toxicity levels, the combination of AICAR and radiation resulted in a greater than additive enhancement of clonogenic kill of PC3 cells, indicated by CI values less than 1. The anti-diabetic drug metformin may sensitize cells to radiation by acting as an AMPK activator. We observed that the enhancing effect of 0.5 mM AICAR on clonogenic killing activity of radiation was similar to that of 5 mM metformin (Figure 2D). The percentage of propidium iodide-stained cells in sub-G_1_, characteristic of apoptosis, was increased by radiation (2 Gy X-rays) and in a concentration-dependent manner by AICAR (Figure 2E and 2F). Furthermore, the pro-apoptotic effect of single agents was enhanced in both LNCaP and PC3 cells by the simultaneous administration of the combination of treatments. Growth of multicellular spheroids composed of LNCaP cells was delayed by irradiation (Figure 3). Radiation-induced growth delay was enhanced by the simultaneous administration of 5 mM AICAR (Figure 3A). On the basis of AUC values (Figure 3B), the combination of radiation treatment and AICAR resulted in greater than additive inhibition of growth. The inhibition of spheroid growth can be observed in representative images of spheroids at the end of the experiment in Figure 3C. The activation of AMPK by AICAR in LNCaP cells, indicated by phosphorylation of ACC, was unaffected by administration of 2 Gy X-rays (Figure 3D).

### Effect of AICAR on cell cycle progression

**Figure 4:**
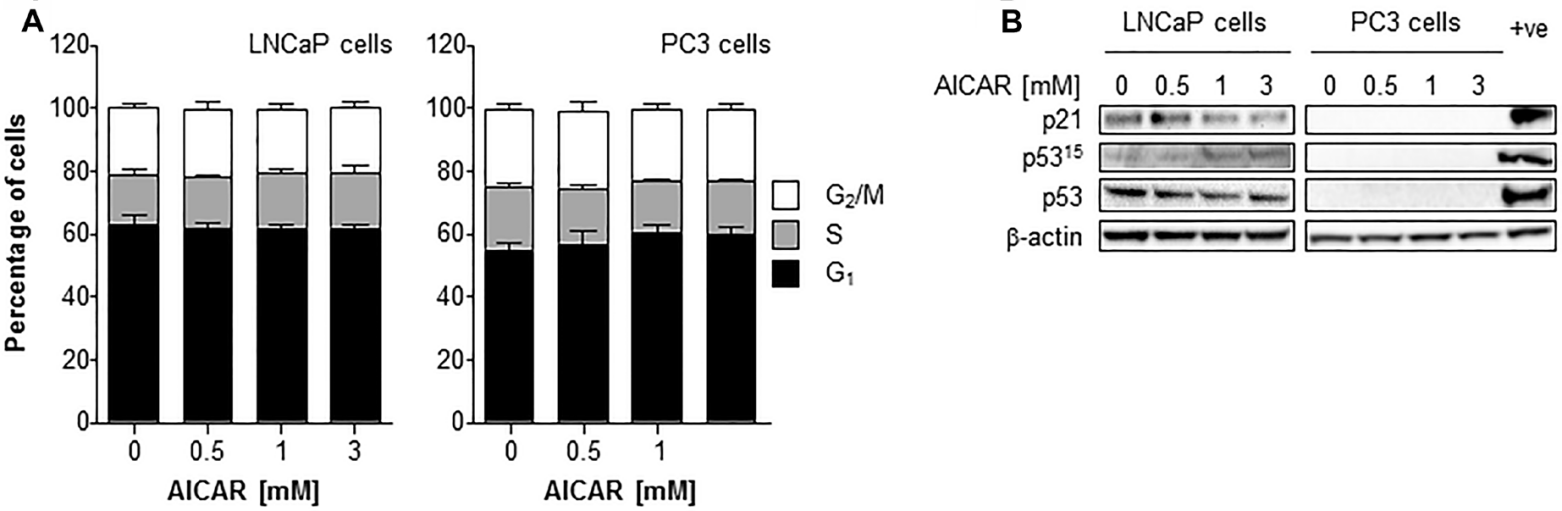
Cell cycle analysis and effect on p53 activity of LNCaP and PC3 cells after treatment with AICAR (**A**) Cell cycle distribution of LNCaP and PC3 cells after 6 h treatment of cells with AICAR. Data are means ± SEM, *n* = 3 (LNCaP cells) and *n* = 4 (PC3 cells). (**B**) Expression of total p53, phosphorylated p53 (serine 15) and p21 in LNCaP and PC3 cells 6 h after treatment. Positive control is LNCaP cells 6 h after irradiation (2 Gy), β-actin is used as a loading control.

The effect of a range of concentrations of AICAR on the cell cycle distribution of asynchronously growing PC3 and LNCaP cells is shown in Figure 4A. AICAR had no significant effect on cell cycle progression 6 h after the initiation of treatment. The effect of AICAR on p53 was also determined 6 h after treatment (Figure 4B). Expression of total or phosphorylated p53 in LNCaP cells was not affected by AICAR. Expression of p21 was used as a marker of p53 transcriptional activity. The failure to increase expression in LNCaP cells indicated no change in p53 activation by AICAR. Expression of p53 (total and phosphorylated) and p21 was not detected in PC3 cells with or without AICAR treatment.

**Figure 5:**
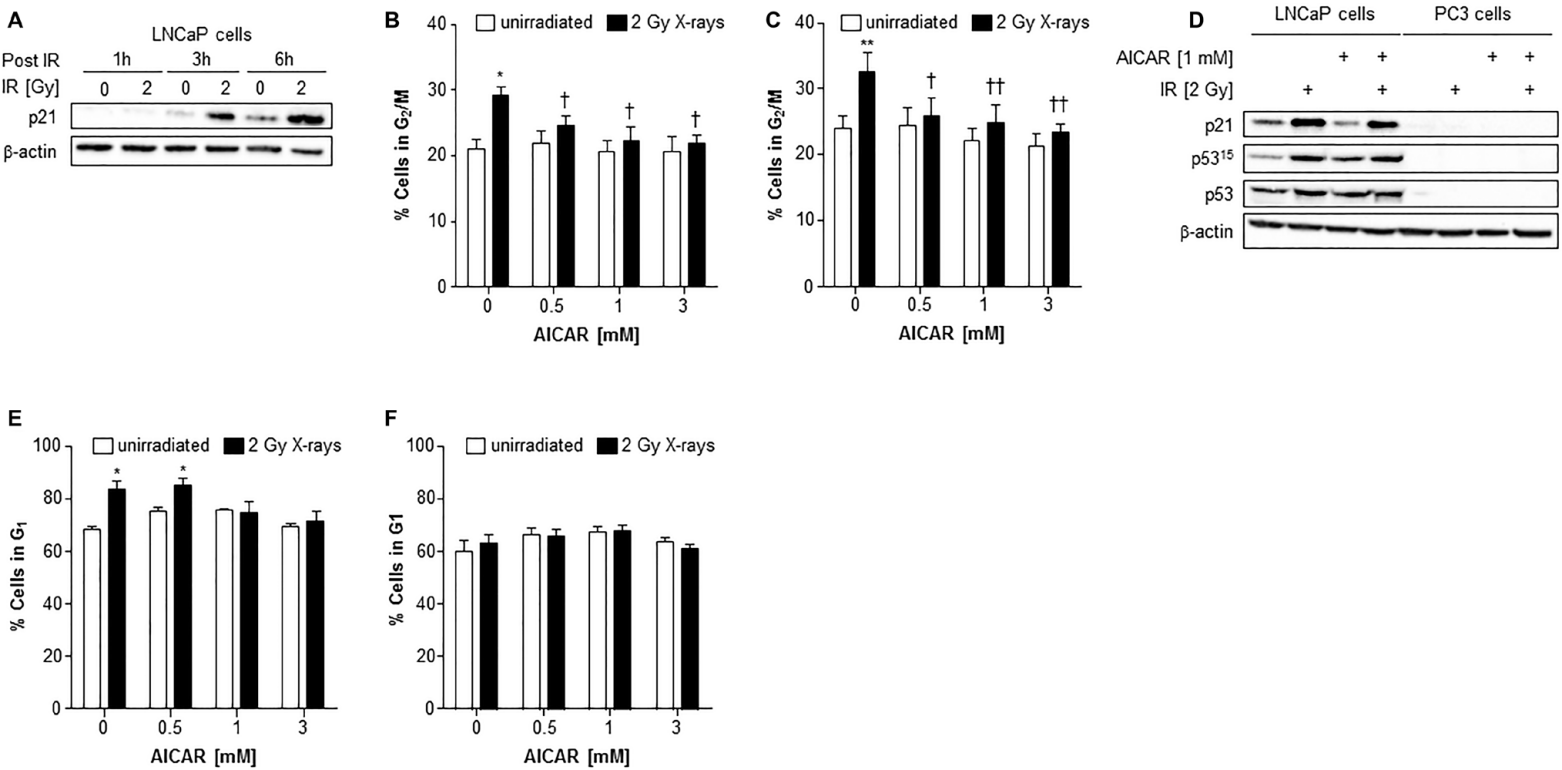
Cell cycle analysis and effect on p53 activity of LNCaP and PC3 cells after combination treatment with AICAR and X-rays (**A**) p21 expression in LNCaP cells 1, 3 and 6 h after irradiation (2 Gy X-rays). Cell cycle analysis after treatment of (**B**) LNCaP cells and (**C**) PC3 cells with radiation and AICAR, alone or in combination. Graphs show proportion of cells in G_2_/M phase of cell cycle 6 h after administration of treatments. (**D**) Total p53, phosphorylated p53 (serine 15) and p21 expression was determined in LNCaP and PC3 cells 6 h after administration of radiation (2 Gy X-rays) or AICAR (1 mM) or combinations of both treatments. β-actin is used as a loading control. (**E**) LNCaP cells and (**F**) PC3 cells in G_1_ phase of cell cycle 24 h after administration of treatments. Data are means ± SEM, *n* = 3 (LNCaP cells) and *n* = 4 (PC3 cells). ^*^p < 0.05 and ^**^p < 0.01 compared to untreated controls, ^†^*p* < 0.05 and ^††^*p* < 0.01 compared to radiation treatment alone.

Irradiation of LNCaP cells induced the expression of p21 after 3 and 6 h administration (Figure 5A). In both LNCaP and PC3 cell lines, radiation alone caused a rise in the G_2_/M population 6 hours after irradiation (Figure 5B and 5C). Although AICAR alone had no significant effect on cell cycle, simultaneous administration of AICAR significantly reduced the radiation-induced cell cycle arrest: the percentage of cells in G_2_/M was similar to those of untreated control cells. Activation of p53, as indicated by phosphorylation of p53 and expression of p21, was observed in LNCaP in response to irradiation (2 Gy X-rays), whereas p53 was not detected in PC3 cells (untreated or irradiated) (Figure 5D). The radiation-induced p53 activation and p21 expression observed in LNCaP cells was not affected by the co-administration of AICAR (1 mM). LNCaP cells which had been irradiated 24 h before fixing and staining with propidium iodide had an increased proportion of cells in G_1_ phase of the cell cycle (Figure 5E). This radiation-induced cell cycle arrest was prevented by simultaneous administration of AICAR (1 and 3 mM). In contrast, in PC3 cells there was no significant arrest in G_1_ cells 24 h after irradiation, and AICAR had no effect on cell cycle (Figure 5F).

### Enhancement of AICAR radiosensitization

**Figure 6:**
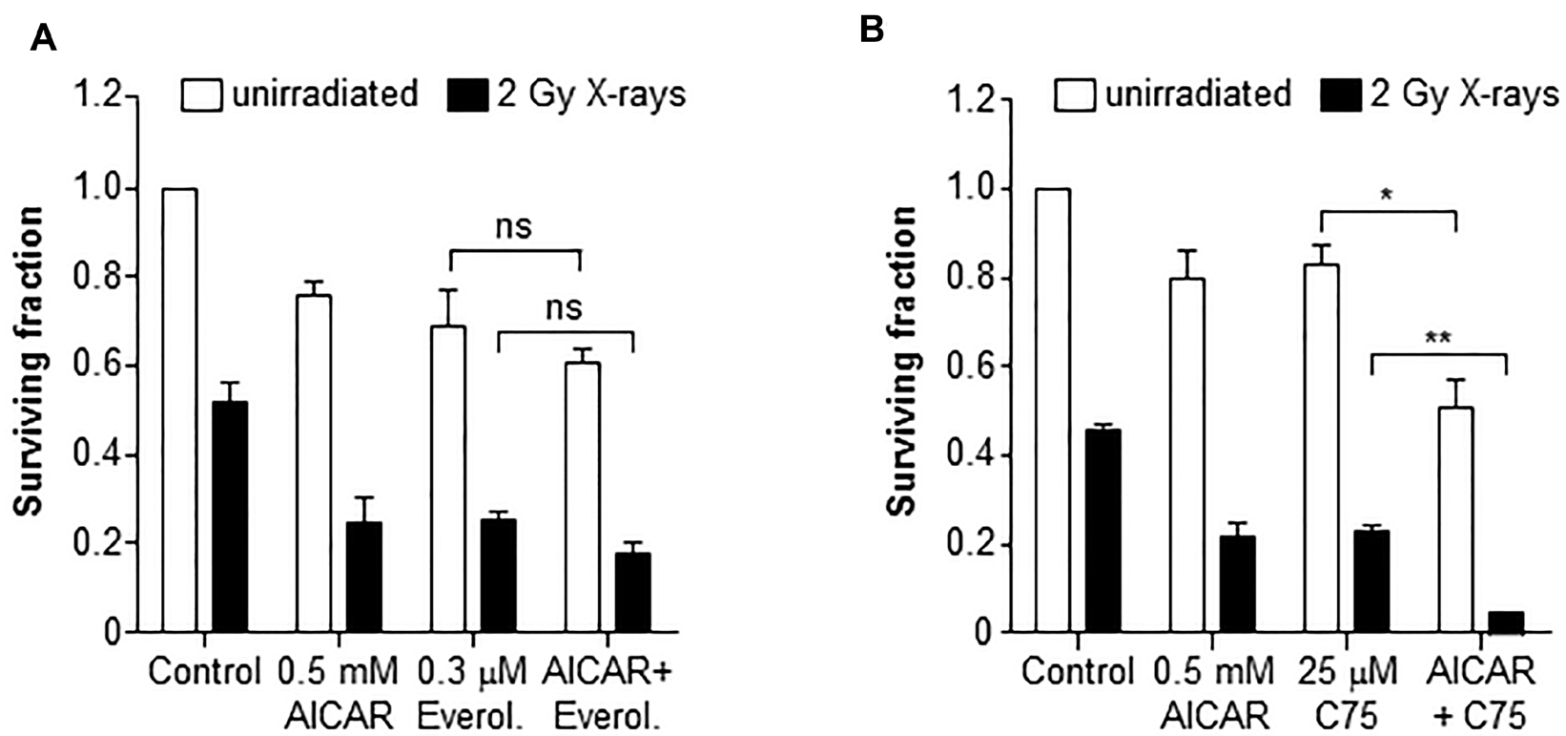
Clonogenic survival of PC3 cells after combinations of AICAR and X-rays with inhibitors of mTOR or FASN Clonogenic assay of PC3 after administration of AICAR (0.5 mM) and (**A**) Everolimus (0.3 mM) or (**B**) C75 (25 µM), singly and in combination, with and without radiation (2 Gy X-rays). Clonogenic assay was carried out 24 h after simultaneous treatment. Data are means ± SEM, *n* = 3. ^*^p < 0.05 and ^**^p < 0.01.

Inhibition of mTOR using everolimus (0.3 µM) enhanced the radiation-induced clonogenic kill of PC3 cells (Figure 6A). However, the co-administration of everolimus and AICAR (0.5 mM) did not significantly increase the clonogenic killing capacity of either agent alone. A triple combination of AICAR, everolimus and X-rays also did not significantly increase the clonogenic kill observed. Conversely, the fatty acid synthase inhibitor C75 enhanced both the radiation-induced and AICAR-induced clonogenic kill of PC3 cells (Figure 6B). Furthermore, a triple combination consisting of C75, AICAR and X-rays had greater clonogenic killing potency than any of the single agents or double combinations (*p* < 0.01).

## DISCUSSION

The AMPK signalling pathway regulates cellular energy processes and is activated during stress conditions. AMPK is a mediator of tumor suppressor pathways and its activation can inhibit the growth of prostate cancer cells [11]. Ionizing radiation may also activate AMPK [9], contributing to its pro-apoptotic and anti-proliferative effect. Furthermore, high expression of AMPKα may associate with improved prognosis [23]. Here, we investigated the radiosensitizing potential of the AMPK activating drug AICAR and compared the response of two prostate cancer cell lines.

The cytotoxicity of AICAR was apparent in two prostate cell lines, PC3 and LNCaP, in two *in vitro* models of tumor growth, clonogenic assays using 2-dimensional cultures and multicellular tumor spheroids using 3-dimensional cultures. The observed cytotoxic and growth inhibitory effects of AICAR occurred at concentrations which increased phosphorylation of acetyl Co-A carboxylase (ACC), indicating that these concentrations of AICAR were sufficient to activate AMPK. The cytotoxicity of many anti-cancer drugs is mediated directly or indirectly by the induction of oxidative stress [24]. Oxidative stress activates AMPK in order to maintain the redox balance of cells [25]. Intriguingly, Kuznetsov *et al.* [26] observed that, although AICAR alone not induce the generation of reactive oxygen species, the antioxidant NAC decreased, but did not prevent, AICAR-induced apoptosis of acute lymphoblastic leukaemia cells 48 h after co-administration. However, we observed that NAC did not prevent the AICAR-induced cytotoxicity of PC3 cells 24 h after administration, indicating that the cytotoxic activity of AICAR is not mediated by oxidative stress.

The biguanide drug metformin has anti-tumor activity [21] and patients taking this drug to treat their type 2 diabetes may have a reduced risk of certain cancers [27], including prostate cancer [28]. Although the cancer killing effect of metformin has been attributed to the indirect activation of AMPK [29], anti-proliferative effects of metformin in LNCaP cells have also been proposed to occur in an AMPK-independent manner [30]. We observed here that, in addition to decreasing survival of clonogens of PC3 prostate cancer cells, metformin enhanced the clonogenic killing activity of ionizing radiation. This is in agreement with previous studies of metformin combined with ionizing radiation [9, 23], which was demonstrated to be dependent on AMPK activation in pancreatic cancer cells [19]. However, the concentrations of metformin used experimentally varies greatly and concentrations required for AMPK activation, anti-proliferative effects and radiosensitization demonstrated here and previously [23, 30, 31] may be 1–2 orders of magnitude higher than those estimated to occur in human plasma following therapeutic doses [17]. Furthermore, the radiosensitizing effect of metformin appears to be cell line dependent and was not always greater than an additive affect [9, 23, 32]. Zanella *et al.* [31] also suggested that the sensitizing effect of metformin with radiotherapy *in vivo* could be caused by re-oxygenation of radio-resistant hypoxic cells rather than an effect on tumor cell radiosensitivity. Here, we calculated dose enhancement ratios (DER) of AICAR from clonogenic assays, and the values obtained indicated a synergistic interaction [33]. The combination index analysis method was also utilized to determine interaction between therapeutic modalities. This involves the treatment of cells with a fixed dose ratio of AICAR and ionizing radiation [34]. Combination indices of less than 1 were observed at all toxicity values (Figure 2C), confirming a synergistic interaction between AICAR and ionizing radiation *in vitro*. The enhancing effect of AICAR on radiation observed in PC3 cells in 2-dimensional clonogenic assays was confirmed in LNCaP cells using 3-dimensional spheroid growth assays, although higher concentrations of AICAR were required to sensitize spheroids. The relative resistance of multicellular spheroids has previously been observed by us [20, 36] and is most likely due to differences in drug penetration and the microenvironment of the various layers within the spheroid [37]. When comparing assays in 2-dimensional cultures, results were comparable for both cell lines.

Although cells are most sensitive to ionizing radiation in the G2/M phase of cell cycle [38], the administration of AICAR as a single treatment did not induce cell cycle arrest and had no effect on cell cycle distribution in either PC3 or LNCaP cells. This may explain why there was no significant benefit in scheduling AICAR before x-irradiation. Simultaneous administration of both treatments was the most cytotoxic schedule. Cell cycle arrest was observed in the G2/M phase after irradiation of both cell lines.

Abnormalities in p53 are not as common in prostate cancer as they are in other cancers, and LNCaP and PC3 cells differ in their p53 status, expressing wild type p53 and non-functional p53, respectively [39]. Following irradiation, the observed cell cycle arrest of PC3 cells in the G2/M phase may occur through a p53-independent mechanism, such as through p53-independent p21 activation as previously shown for ionizing radiation in the same cell line [9]. However, we observed that p21 expression was not detected in PC3 cells in response to irradiation. The abrogation of radiation-induced cell cycle arrest by AICAR in both cell lines, using concentrations and timepoints shown to radiosensitize, indicates that this may be one mechanism whereby AMPK activation may lead to unrepaired DNA damage and mitotic catastrophe, resulting in cell death [40]. The G1 arrest observed in LNCaP cells, but not PC3 cells, 24 h after irradiation was most likely due to the presence of functional p53 in LNCaP, but not PC3, cells. The suppression of radiation-induced G1 arrest by AICAR in LNCaP cells may be another mechanism contributing to radiosensitization in these cells.

Loss of phosphatase and tensin homologue (PTEN) is the most common mutation of a tumor suppressor gene in prostate cancer, and both cell lines used in this study have inactive PTEN [41]. PTEN is a negative regulator of the PI3K-Akt pathway and downstream mTOR signalling and its loss of function leads to dysregulation of this pathway, increasing proliferation, survival, resistance to therapy and altering metabolism [42]. Re-introduction of PTEN in PTEN-null prostate cancer or inhibition of the PI3K pathway also decreased expression of fatty acid synthase [43], demonstrating the importance of its role in prostate cancer. Radiation-induced activation of the PI3K/Akt/mTOR pathway may limit the effectiveness of radiotherapy and inhibition of this pro-survival pathway enhanced sensitivity to radiotherapy in glioblastoma and prostate cancer cells [44, 45].

It has been suggested that drugs which inhibit mTOR signalling may increase the efficacy of other chemotherapeutic agents [21]. We observed that the mTOR inhibitor everolimus enhanced the clonogenic killing activity of X-rays in PC3 cells. Sengupta *et al.* [46] demonstrated that the mTOR inhibitor rapamycin increased the anti-proliferative activity of AICAR in leukaemia cells. However, we observed that a combination of everolimus and AICAR induced only a slight, non-significant, increase in clonogenic killing activity compared to either agent alone. Moreover, the simultaneous administration of AICAR and everolimus did not enhance the radiosensitization caused by either drug alone. AMPK activation by AICAR suppresses the mTOR signalling pathway [47], leading to decreased proliferation and survival. Thus, it is possible that administration of AICAR alone is sufficient to abrogate the mTOR hyperactivity in PC3 cells caused by inactive PTEN, making addition of an mTOR inhibitor redundant.

AMPK phosphorylates and inactivates ACC [48], the most highly regulated enzyme in the fatty acid synthase pathway. We also observed the phosphorylation of ACC in response to AICAR at concentrations used in this study. Furthermore, metformin decreased FASN protein levels in breast cancer cells [49] and the FASN inhibitor orlistat up-regulated AMPK activity in lung cancer cells [50]. This demonstrates the critical role of fatty acid metabolism in cellular energy production and suggests an important interaction between AMPK and FASN. We have previously showed that the fatty acid synthase inhibitor C75 sensitized prostate cancer cells to ionizing radiation [20]. Although the potential use of C75 has been limited due to side effects of appetite suppression and weight loss [51], this may be overcome using enantiomers of C75 which retain anti-tumor and radiosensitizing activity in the absence of anorexia [35, 52]. We demonstrate here that C75 is able to enhance the clonogenic killing activity of AICAR. This suggests that combining agents which target two important pathways in prostate cancer carcinogenesis, lipogenesis and energy regulation, is a promising therapeutic approach. Furthermore, the triple combination of C75, AICAR and x-irradiation induced a level of clonogenic kill greater than the anticipated additive effect of any combination of two modalities. This indicates that drugs which target pathways which are dysregulated in aggressive cancers and are associated with altered metabolism, proliferation, and resistance to therapy can increase the efficacy of radiotherapy.

In conclusion, we have demonstrated that activation of AMPK using AICAR enhanced the clonogenic killing and spheroid growth delay effect of x-irradiation in prostate cancer cell lines. The interaction appeared to be synergistic and the mechanism of interaction may involve alterations in cell cycle regulation, regardless of the p53 status of the cells. Although this study suggests the potential use of activators of AMPK in combination with radiotherapy of prostate cancer cells, AICAR has limited oral bioavailability [53]. Therefore, the use of novel AMPK activators with greater specificity and bioavailability are currently being developed [54]. Moreover, the radiosensitizing activity of AMPK activators may be most beneficial in the management of advanced disease where they can be combined with molecular targeted radiopharmaceuticals, such as those binding prostate specific membrane antigen (PSMA) [55].

## MATERIALS AND METHODS

### Reagents

All cell culture media and supplements were purchased from Life Technologies (UK), unless stated otherwise. AICAR was purchased from Santa Cruz Biotechnology (Germany). All other chemicals were from Sigma-Aldrich (UK). Stock solutions of AICAR were prepared in dimethyl sulfoxide (DMSO). Control treatments contained DMSO alone in culture medium.

### Tissue culture

Human prostate cancer cell lines, PC3 and LNCaP, were obtained from American Type Culture Collection (Manassas, VA, USA) and were used in this study for less than 6 months after resuscitation. PC3 cells were maintained in F12K medium supplemented with 10% (*v/v*) fetal bovine serum (Labtech, UK), 2 mM L-glutamine, 0.1 mM sodium pyruvate and 50 μg/ml gentamicin. LNCaP cells were maintained in RPMI 1640 medium supplemented with 10% (*v/v*) fetal bovine serum (Hyclone, ThermoFisher Scientific, UK), 1% (*v/v*) HEPES, 1% (*v/v*) D-glucose, 1 mM sodium pyruvate, 4 mM L-glutamine, 50 μg/ml gentamicin.

### MTT toxicity assay

MTT reduction was performed according to the method of Mosmann [56]. Cells were seeded in 96-well plates and incubated for 2 days to allow exponential phase growth. Cells were then washed with PBS and medium containing drug at the required concentration was added. After 24 h incubation, MTT was added to a final concentration of 0.5 mg/ml and cultures were incubated for 2 h. Cells were then solubilized with DMSO before measuring absorbance at 570 nm.

### Clonogenic survival assay

PC3 cells were seeded in 25 cm^2^ flasks at 10^5^ cells/flask. When cultures were in exponential growth phase, medium was removed and replaced with fresh medium containing drug. Cells were then incubated for 24 h at 37° C in 5% CO_2_. For the determination of optimal sequencing of therapeutic agents, three different combination treatment schedules were assessed: (i) radiation and AICAR administered simultaneously, (ii) radiation administered 24 h before AICAR, (iii) radiation administered 24 h after AICAR. After treatment, cells were seeded for clonogenic survival assay as previously described [20, 35]. Cells were incubated at 37° C in 5% CO_2_ for 13 days. Colonies were fixed in methanol, stained with crystal violet solution and colonies of at least 50 cells were counted. To assess the role of reactive oxygen species in AICAR-induced cytotoxicity, cells were co-incubated with the antioxidant N-acetyl cysteine (NAC, 1 mM).

Cells were irradiated using an X-Strahl RS225 x-ray irradiator at a dose rate of 1.6 Gy per minute. The cytotoxic interaction between AICAR and x-radiation *in vitro* was assessed according to the method of Chou and Talalay [34], which is based on the median-effect principle. Briefly, clonogenic assay was carried out using a fixed dose ratio of drug to radiation, based on the concentrations required to kill 50% of clonogens (IC_50_) of each single agent, so that the proportional contribution of each agent in the mixtures was the same at all treatment intensities. The effectiveness of combinations was quantified by calculating a combination index (CI) at various levels of cytotoxicity. CI < 1, CI = 1 and CI > 1 indicate synergism, additivity and antagonism, respectively.

### Multicellular spheroid growth assay

Multicellular tumor spheroids consisting of LNCaP cells were obtained using the liquid overlay technique [57]. Spheroids were initiated by inoculating cells into a plastic flask coated with 1% (*w/v*) agar. After 3 days, aliquots of spheroids were transferred to sterile plastic tubes and centrifuged at 12 × g for 3 min. Thereafter, spheroids were re-suspended in serum-free culture medium containing AICAR or irradiated. After treatment, spheroids of approximately 100 µm in diameter were transferred individually into agar-coated wells of 24-well plates, as previously described [58]. Individual spheroid growth was monitored twice per week for three weeks using an inverted phase-contrast microscope connected to an image acquisition system. Two perpendicular diameters, d_max_ and d_min_, were measured using image analysis software (ImageJ) and the volume, V (μm^3^), was calculated using the formula: V = π × d_max_ × d_min_^2^ / 6. The area under the V/V_0_ versus time curve (AUC) was calculated for individual spheroids using trapezoidal approximation.

### Cell cycle analysis

Following AICAR treatment for 6 or 24 h, LNCaP or PC3 cells were trypsinized, then washed twice with PBS. Cells were fixed by treatment with ice cold 70% (v/v) ethanol; then washed twice with PBS and re-suspended in PBS containing propidium iodide (10 μg/ml) and RNase A (200 μg/ml). Cells were stained for 30 min before flow cytometric analysis using a FACSVerse analyser (BD Biosciences, UK). Data were analysed using FlowJo software.

### Immunoblotting

Antibodies against β-actin were obtained from Abcam, UK. The antibody against p53 was obtained from Santa Cruz Biotechnology, Germany. Antibodies against p53 phosphorylated at serine 15 was obtained from New England Biolabs, UK. The antibody against p21 was obtained from BD Biosciences, UK. Antibody against phosphorylated acetyl Co-A was obtained from Merck, UK. Whole cellular protein extracts were resolved in reducing and denaturing conditions by sodium dodecyl sulphate polyacrylamide gel electrophoresis. Proteins were transferred on to polyvinylidene difluoride (PVDF) Immobilon-P membranes (Merck, UK). Membranes were blocked with 7.5% (*w/v*) milk for 2 h prior to incubation with the primary antibodies overnight at 4° C. Membranes were then washed and incubated at room temperature for 1 h with horseradish peroxidase-conjugated secondary anti-mouse or anti-rabbit antibody (Santa Cruz Biotechnology, Germany) to enable chemiluminescent detection using ECL (ThermoFisher Scientific, UK).

### Statistical analysis

Data are presented as means ± standard error of the mean (SEM), with the number of independent repetitions provided in the legend to each figure. Statistical significance was determined using Student’s *t* test. A *P* value < 0.05 was considered to be statistically significant and < 0.01 highly significant.
